# Surgical accuracy of open platform image-based robotic-assisted total hip arthroplasty

**DOI:** 10.1186/s42836-026-00370-1

**Published:** 2026-02-06

**Authors:** Wang-Fung Rex Mak, Yuan Zhang, Jiying Chen, Jonathan Patrick Ng, Cham-Kit Wong, Gloria Yan-Ting Lam, Tsz Lung Choi, Wei Chai, Patrick Shu-Hang Yung, Zongke Zhou, Michael Tim-Yun Ong

**Affiliations:** 1 Prince of Wales Hospital, Hong Kong SAR, China; 2 Xinqiao Hospital, Army Medical University, Chongqing, 400038 China; 3 The First Medical Centre, Chinese PLA General Hospital, Beijing, 100853 China; 4 Alice Ho Miu Ling Nethersole Hospital, Hong Kong SAR, China; 5 Fourth Medical Centre of PLA General Hospital, Beijing, 100089 China; 6 Chinese University of Hong Kong, Hong Kong SAR, China; 7 CUHK Medical Centre, Hong Kong SAR, China; 8 West China Hospital, Orthopedic Research Institute, Sichuan University, Chengdu, 610041 China

## Abstract

**Background:**

Precise acetabular cup positioning is critical for the success and longevity of total hip arthroplasty (THA). Robotic-assisted systems enhance placement accuracy, with closed-platform systems being well-established. A pertinent question is whether newer open-platform systems, which offer implant flexibility, achieve comparable accuracy. This study evaluates the radiographic accuracy of a novel, open-platform robotic system (Yuanhua KUNWU) in achieving the planned acetabular component position.

**Methods:**

A multi-centre retrospective review of 87 consecutive primary robotic THA procedures performed using the KUNWU system was conducted. Pre-operative CT-based planning defined the target acetabular inclination (AI) and anteversion (AV). The primary outcome was the deviation between the planned position and the post-operative CT-measured position. Secondary outcomes included the proportion of cups within the Lewinnek and Callanan safe zones and the accuracy of leg length and offset restoration.

**Results:**

The mean deviation from the planned position to the post-operative CT was −2.7° for inclination (95% CI: −3.7° to −1.8°, *P* < 0.001) and 1.0° for anteversion (*P* = 0.058). Overall, 80.5% (70/87) of cups were placed within the combined Lewinnek and Callanan safe zones. A significant difference was found in combined offset (mean 2.79 mm, *P* = 0.002) but not in leg length discrepancy (*P* = 0.302). Interobserver reliability was excellent for all measurements.

**Conclusion:**

The KUNWU open-platform robotic system facilitates accurate and reliable acetabular cup positioning, with minimal deviations from the pre-operative plan and a high rate of placement within the classic safe zones. These results support its use as a precise tool for component positioning in THA.

## Introduction

Total hip arthroplasty (THA) is a common and effective surgical procedure [1]. Proper acetabular cup positioning is crucial for long-term survivorship and success of the surgery. Mal-positioning of the acetabular cup may cause altered hip biomechanics, leading to instability, dislocation, polyethylene wear, osteolysis, or even leg length discrepancy (LLD) [2, 3]. In metal-on-metal bearing surfaces, eccentric loading may precipitate metallosis, and in ceramic-bearing surfaces, it can contribute to squeaking, stripe wear, or fracture. Consequently, the accurate placement of the acetabular component during THA remains a paramount yet challenging surgical objective.

The concept of a “safe zone” for acetabular orientation was popularised by Lewinnek et al., who defined it as 30–50° inclination and 5–25° anteversion [4]. A subsequent study by Callanan et al. proposed a more refined zone of 30–45° inclination and 5–25° anteversion [5]. However, a substantial proportion of dislocations occur even with cups placed within these radiographic boundaries [6, 7], highlighting their limitations. This has led to increased emphasis on individual patient factors, particularly spinopelvic mobility and balance, as critical determinants of stability. Therefore, while meticulous pre-operative planning is essential, the accurate execution of that plan is arguably even more crucial.

Robotic-assisted THA systems have emerged as a valuable technology to enhance the precision of acetabular reaming and component positioning compared to conventional manual techniques. These systems are broadly categorized into closed-platform and open-platform architectures. Closed-platform systems are proprietary and restrict the surgeon to a single manufacturer’s implants, while open-platform systems offer flexibility by accommodating implants from multiple manufacturers. A pertinent question is whether the accuracy of newer open-platform systems is comparable to that of established, implant-specific closed-platform systems [8].

**Fig. 1:**
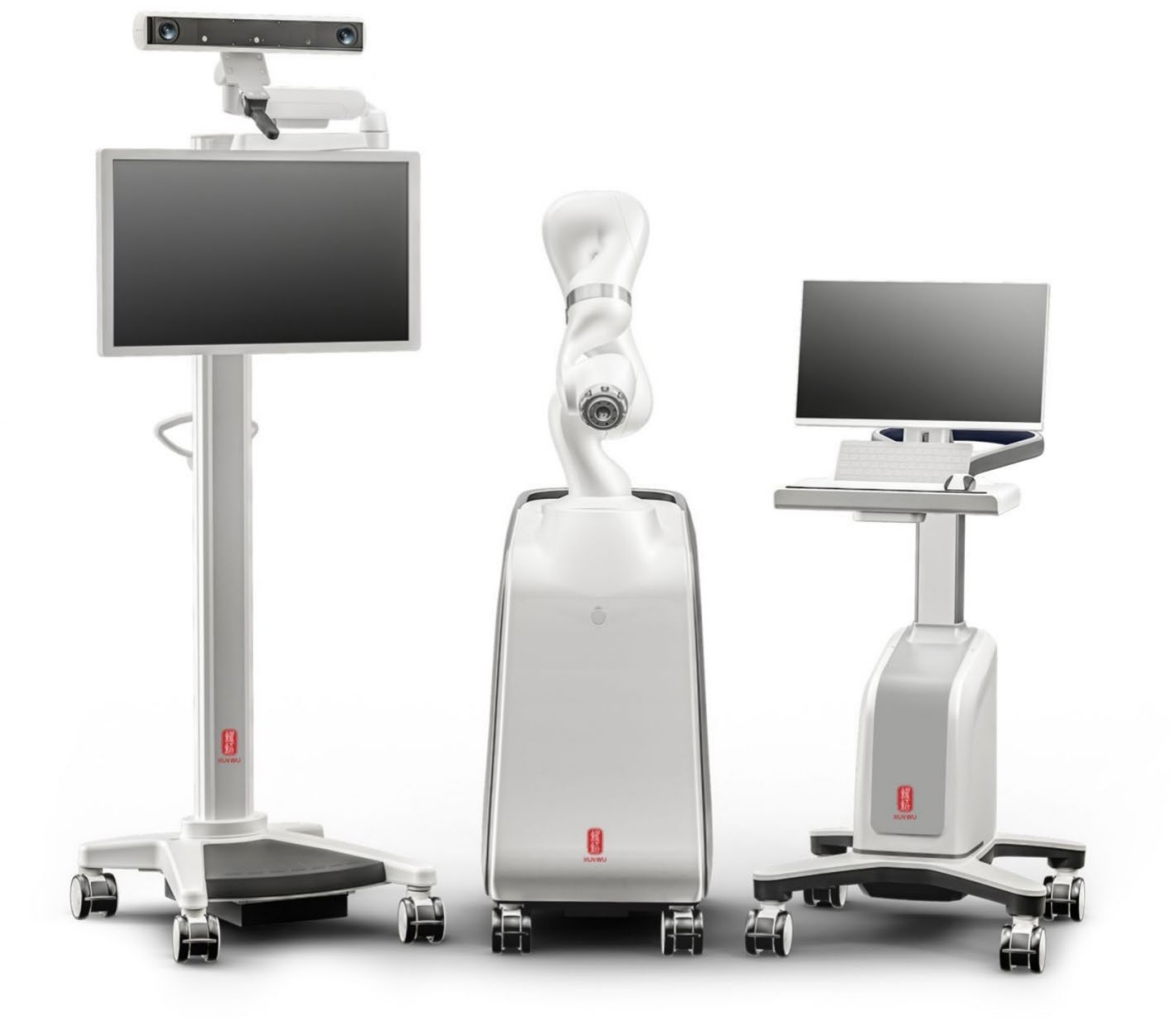
Graphic showing the Kunwu robotic system

The Yuanhua KUNWU system (Yuanhua Robotics, Hong Kong, China) is a semi-active, image-based, open-platform robotic arm with 7 degrees of freedom (Fig. 1). Its open architecture allows for the use of implants from multiple manufacturers. The surgical workflow begins with a pre-operative CT scan, and implant positioning is planned with the system software based on the patient’s anatomy and the surgeon’s preference. Intra-operatively, after initial surgical exposure, a surface registration process is performed to match the patient’s anatomy to the pre-operative imaging data. The robotic arm then guides the orientation and depth of acetabular reaming. Finally, the acetabular component is attached to the robotic arm and implanted under precise robotic guidance. The final cup position is confirmed by digitizing 5 co-planar points on the face of the implanted cup using a handheld probe.

This study presents a case series evaluating the accuracy of this novel open-platform, image-based robotic system in achieving planned acetabular component position in THA.

## Methods

### Study design

This study constitutes a retrospective analysis of prospectively collected data in the authors’ institutional joint registry. Ethics approval was obtained from the Institutional Ethics Review Committee of the Joint CUHK-NTEC Clinical Research Ethics Committee.

**Fig. 2:**
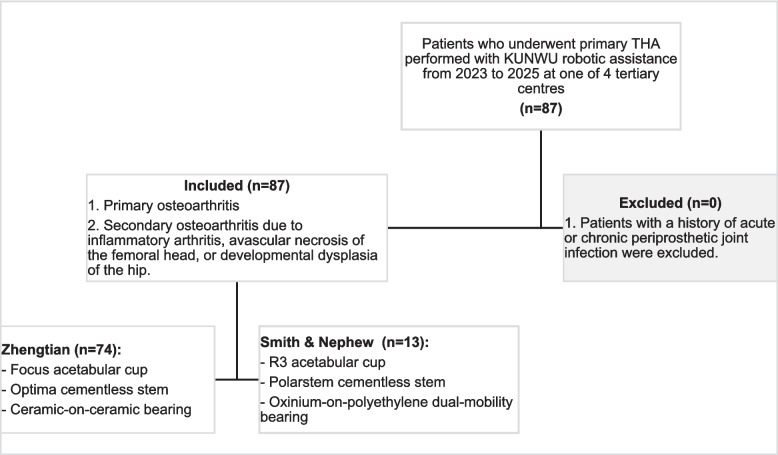
Inclusion and exclusion flowchart

Patients who underwent primary THA performed with KUNWU robotic assistance from 2023 to 2025 at one of 4 tertiary centres were included. The primary indications for surgery were primary osteoarthritis as well as secondary osteoarthritis due to inflammatory arthritis, avascular necrosis of the femoral head, or developmental dysplasia of the hip. Patients with a history of acute or chronic periprosthetic joint infection were excluded. Acetabular cup placement and alignment were planned according to the surgeon’s preference. The choice of implant was based on surgeon preference and availability (Fig. 2). All procedures were performed by a total of 6 surgeons, all of whom were high-volume arthroplasty surgeons experienced with specialists in robotic hip arthroplasties and specifically trained on the KUNWU system. Clinical data collected included patient demographics, operative details, and inpatient records.

### Radiological assessment

**Fig. 3:**
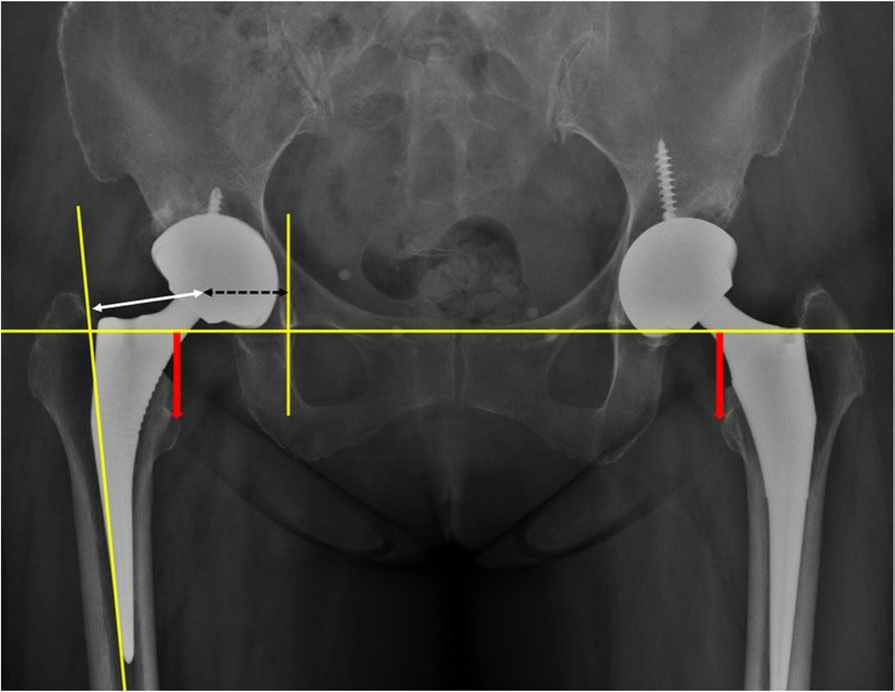
Radiographical measurement of LLD (thick red arrow), femoral offset (thin white arrow), acetabular offset (black dotted arrow), and combined offset (sum of femoral and acetabular offset)

Post-operatively, an anterior–posterior (AP) radiograph of the pelvis and a lateral radiograph of the hip were obtained. A computed tomography (CT) scan was performed to assess the discrepancy between planned and actual cup positions, in particular the acetabular version (AV) and inclination (AI). CT measurements were performed using Mimics 24.0 (Materialise, Leuven, Belgium) according to the definitions established by Murray [9]. On AP pelvic radiographs, leg length discrepancy (LLD) was defined as the difference in distances between the bilateral teardrops and the bilateral lesser trochanters. Acetabular offset was defined as the horizontal distance from the radiographic teardrop to the centre of rotation (COR) of the hip; femoral offset was defined as the distance from the COR to the femoral canal axis; and combined offset was defined as the sum of acetabular and femoral offset (Fig. 3).

### Statistical analysis

The degree of deviation between planned, intra-operative (via robotic probe), as well as post-operative (via imaging) cup position was recorded and compared using paired samples Wilcoxon signed rank test or paired samples T-test. Radiological outliers were defined as those outside of the safe zone of Lewinnek and/or Callanan. Any difference in planned and post-operative leg length discrepancy, as well as combined offset were also analysed. To ensure measurement reliability, two independent investigators performed the radiological measurements. Interobserver reliability was evaluated by intra-class correlation (ICC) and Cronbach’s alpha values. Statistical analysis was performed using IBM SPSS version 28 (Armonk, NY: IBM Corp), with a *P*-value < 0.05 considered statistically significant.

## Results

There was a total of 87 patients who underwent KUNWU robotic-assisted THA (Table 1). All surgeries utilised cementless fixation for the acetabular cup and femoral stem. 74 (85%) of those used Zhengtian (Focus acetabular cup, Optima cementless stem, ceramic-on-ceramic bearing), and 13 (15%) used Smith & Nephew (R3 acetabular cup, Polarstem cementless stem, oxinium-on-polyethylene dual-mobility bearing). The mean age of the patients was 58.1 ± 12.1 (1SD) years old, of which 45.9% were male. Mean BMI was 24.5 ± 3.7 (1SD). The most common indication for surgery was avascular necrosis of the hip (50.6%), followed by primary osteoarthritis (28.7%) and developmental dysplasia (11.5%). There were no post-operative complications, including wound infection, neurovascular injury, or venous thromboembolism, recorded during the inpatient stay.

**Table 1:** Demographics of the cohort

Subjects (*n*)	87
Age [(years, mean (SD)]	58.3 (12.1)
Gender (Male/Female)	40/47
Diagnosis (OA/AVN/DDH/Others)	25/44/10/7
Operative side (left/right)	44/43
OT Time [(minutes, mean (SD)]	112.4 (25.9)
BMI [mean (SD)]	24.5 (3.7)

The interobserver reliability analysis demonstrated excellent agreement between the two raters across all measured parameters (Table 2). A comparison between pre-operative planning and intra-operative robotic verification showed a statistically significant difference for both AI (mean difference −1.8°, 95% CI −2.3– −1.2°, *P* < 0.001) as well as AV (mean difference 0.9°, 95% CI 0.3–1.5°, *P* = 0.002) (Table 3). When comparing the pre-operative plan to the post-operative CT scan (Table 4), the difference remained significant for AI (mean difference −2.7°, 95% CI −3.7– −1.8°, *P* < 0.001) but not AV (mean difference 1.0, *P* = 0.058). There was a significant difference in the achieved versus planned combined offset (mean difference 2.79 mm, *P* = 0.002) but not LLD (*P* = 0.302) (Table 5).

**Table 2:** Intra-class reliability analysis between two raters

Measurement	Cronbach’s α	ICC	95%CI lower	95%CI upper
Anteversion	0.980	0.962	0.942	0.975
Inclination	0.967	0.937	0.906	0.959
Leg Length	0.990	0.980	0.970	0.987
Combined Offset	0.972	0.945	0.918	0.964

**Table 3:** Comparison of alignment between pre-operative planning and intra-operative verification

Indicators	Anteversion	Inclination
Planned (SD)	20.0 (2.20)	40.2 (1.1)
Intra-op Assessment (SD)	20.9 (3.6)	38.4 (2.4)
Mean Difference (SD) (Intra-op-Planned)	0.9 (3.0)	−1.8 (2.6)
95%CI lower	0.3	−2.3
95%CI upper	1.5	−1.2
T/Z	−3.165	−6.338
P	0.002	< 0.001

**Table 4:** Comparison of alignment between pre-operative planning and post-operative imaging

Indicators (*n* = 89)	Anteversion	Inclination
Planned (SD)	20.0 (2.20)	40.2 (1.1)
Post-op Measurement (SD)	21.0 (4.8)	37.4 (4.4)
Mean Difference (SD) (Measurement-Planned)	1.0 (5.0)	−2.7 (4.6)
95%CI lower	0.0	−3.7
95%CI upper	2.1	−1.8
T/Z	1.918	−5.451
P	0.058	< 0.001

**Table 5:** Comparison of LLD and combined offset

	**LLD**	**Combined offset**
Intra-op Assessment (SD)	2.19 (6.48)	−0.78 (6.03)
Post-op Measurement (SD)	1.42 (9.23)	1.96 (5.73)
Mean Difference (SD) (Plan-Measurement)	−0.77 (5.40)	2.74 (7.31)
95%CI lower	−1.92	1.18
95%CI upper	0.38	4.30
T/Z	−1.332	3.495
P	0.186	0.001

**Fig. 4:**
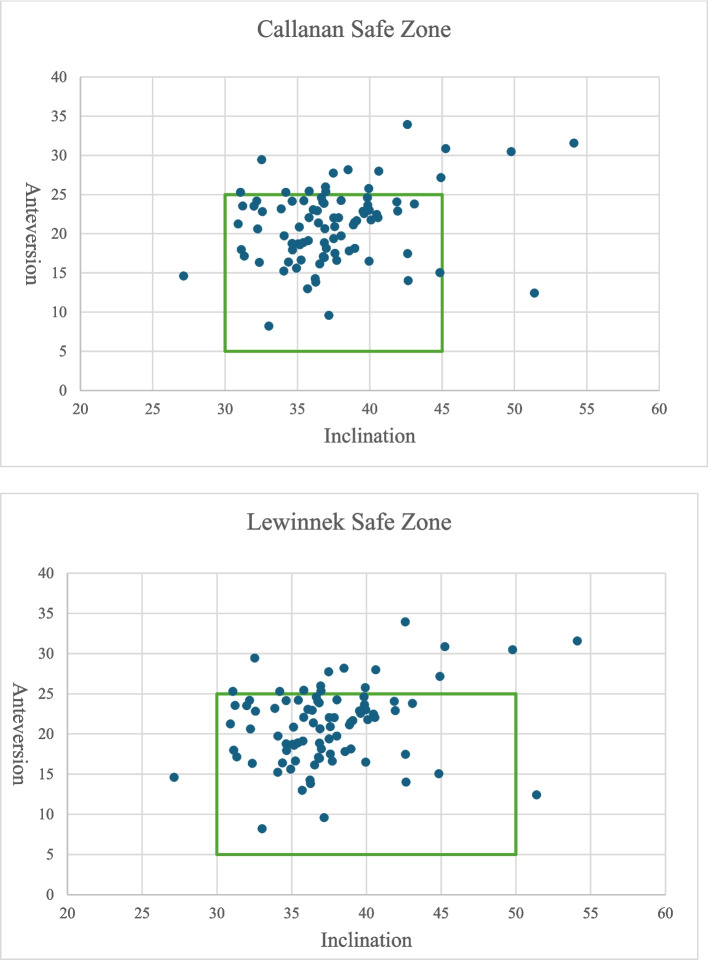
Scatter chart showing alignment of all THAs within safe zones (green box)

Regarding radiological outliers, 82.8% (72/87) of all hips achieved AV within both Callanan and Lewinnek safe zones, 94.3% (82/87) for AI, and 80.5% (70/87) of all cases were within both safe zones for both AI and AV. (Table 6, Fig. 4).

**Table 6:** Percentage of THAs within radiological safe zones of Callanan and Lewinnek

Safe Zone	Anteversion	Inclination	Anteversion & Inclination
Callanan Safe Zone (n/n, %)	72/87, 82.8%	82/87, 94.3%	70/87, 80.5%
Lewinnek Safe Zone (n/n, %)	72/87, 82.8%	84/87, 96.6%	70/87, 80.5%

Subgroup analyses were performed based on comorbidities and implant manufacturer (Tables 7 and 8). The two groups were comparable in terms of BMI and medical comorbidities. When comparing intra-operative verification to the pre-op plan, the ZT (Zhengtian) group showed a significant change in both AV (Mean = +1.39°, *P* < 0.001) and AI (Mean = −2.04°, *P* < 0.001). The S&N (Smith & Nephew) group showed a significant decrease in AV (Mean = −1.82°, *P* = 0.029) but not in AI (Mean = −0.61°, *P* = 0.344). When comparing post-operative CT to the pre-op plan, the ZT group showed a significant difference in both AV (Mean = +1.68°, *P* = 0.003) and AI (Mean = −2.84°, *P* < 0.001). The S&N group showed a decrease in both AV (Mean = −2.70°, *P* = 0.101) and AI (Mean = −2.59°, *P* = 0.052) that did not reach statistical significance, likely due to the smaller sample size. To summarise, the ZT group showed consistent, statistically significant deviations between planned alignment and both intra-op and post-op measurements. The S&N group showed a significant difference in only one parameter (AV intra-op vs. pre-op), though a consistent decreasing trend was observed across the parameters. Importantly, the absolute mean degree of deviation in all groups was less than 3°.

**Table 7:** Subgroup analysis of comorbidities and BMI between implants

Co-morbidities [% (n)]	Zhengtian Group	Smith&Nephew Group	*P*-value
Pulmonary disease	9.5% (7/74)	15.4% (2/13)	0.618
Hypertension	29.7% (22/74)	53.8% (7/13)	0.114
Chronic kidney disease	8.1% (6/74)	0% (0/13)	0.585
Diabetes Mellitus	5.4% (4/74)	15.4% (2/13)	0.218
Cardiovascular disease	5.4% (4/74)	15.4% (2/13)	0.218
Liver disease	16.2 (12/74)	7.7% (1/13)	0.681
BMI ((SD)kg/m^2^)	24.39(3.73)	24.98(3.39)	0.725

**Table 8:** Subgroup analysis of AV and AI between implants

			**Zhengtian Group**	**Smith & Nephew Group**
**Intra-op – Planned**	AV	Mean (SD)	1.39(2.79)	−1.82(2.65)
		95% CI Lower	0.75	−3.43
		95% CI Upper	2.04	−0.22
		T	4.29	−2.48
		P	< 0.001	0.029
	AI	Mean (SD)	−2.04(2.54)	−0.61(2.22)
		95% CI Lower	−2.63	−1.95
		95% CI Upper	−1.45	0.74
		T	−6.89	−0.99
		P	< 0.001	0.344
**Post-op – Planned**	AV	Mean (SD)	1.68(4.63)	−2.70(5.50)
		95% CI Lower	0.61	−6.03
		95% CI Upper	2.75	0.62
		T	3.12	−1.77
		P	0.003	0.101
	AI	Mean (SD)	−2.84(4.51)	−2.59(4.32)
		95% CI Lower	−3.89	−5.20
		95% CI Upper	−1.79	0.02
		T	−5.41	−2.16
		P	< 0.001	0.052

## Discussion

This study presents the first clinical series on acetabular cup positioning accuracy using a novel open-platform robotic system for THA. Our results demonstrate that the KUNWU system facilitates accurate and reliable component placement, with 80.5% of cups positioned within the combined Lewinnek and Callanan safe zones and mean deviations from plan of less than 3°. The critical importance of precise implant positioning in avoiding complications like dislocation and wear is well-established [2, 4, 10]. In response, computer-navigated and robotic systems have been developed to improve the accuracy of acetabular version, inclination, and limb length restoration [11, 12]. Systematic reviews confirm improved functional scores and radiographic precision with robotic assistance compared to manual THA, though differences in complication rates and mid-term survivorship remain to be proven [13].

The observed deviations, though statistically significant, are small in magnitude and likely within the margin of clinical acceptability. The significant difference in horizontal offset (2.79 mm) is also a small absolute value, and its clinical impact is probably negligible. The high concordance between intra-operative robotic verification and post-operative CT scans validates the system’s feedback mechanism as a reliable real-world check.

Our findings align with the existing literature on robotic THA accuracy. Singh et al., evaluating the closed-platform MAKO system, reported accurate replication of planned inclination but a significant deviation in anteversion (planned 19.4° vs. post-op 28.7°), which they attributed to functional changes in pelvic tilt [14]. In our series, the mean deviations for AI (−2.7°) and AV (1.0°) were notably smaller and more controlled, suggesting the KUNWU system may effectively compensate for such factors or that the planning software incorporates different assumptions. This comparison, while indirect, is favourable for the open-platform system. Similarly, Tian et al. reported that a novel robotic system significantly increased the rate of cup placement within the safe zone compared to the conventional technique (80.85% vs. 50.98%) [15], a result nearly identical to our overall safe zone achievement rate of 80.5%.

The subgroup analysis revealing differences between implant brands is a critical and novel finding. The consistent, significant deviations in the larger ZT group suggest a potential systematic error, possibly related to the specific implant geometry, its interface with the robotic arm, or the planning algorithm for that implant. The trend in the smaller S&N group, while not statistically significant, indicates a different pattern of deviation. This underscores a central challenge and opportunity for open-platform systems: each implant design may require specific calibration to achieve optimal accuracy. This “implant-specific profiling” is inherent to closed-platform systems but must be consciously developed for open platforms. Our study is the first to highlight this need empirically, suggesting that open-platform does not simply mean universal compatibility without further validation for each component.

This pilot study has several limitations. Its retrospective nature and relatively small sample size, particularly in the S&N subgroup, limit the power of the analysis. The lack of patient-reported outcome measures (PROMs) and short-term follow-up preclude any assessment of clinical correlation or long-term survivorship. The involvement of multiple surgeons, while enhancing generalizability, may introduce technique variation. Crucially, the substantial size disparity between implant groups (Zhengtian outnumbered S&N by 4:1) precludes definitive conclusions from the subgroup analysis, though the findings generate an important hypothesis for future study.

Future research should directly compare the accuracy of this open-platform system against other established robotic systems (both open and closed-platform) in a randomized controlled trial. Studies with larger, matched cohorts for each implant type are needed to definitively investigate implant-specific accuracy. Furthermore, research must move beyond static radiographic positioning to evaluate functional implant alignment relative to the hip-spine relationship and its impact on stability and wear. Finally, incorporating PROMs and long-term follow-up will be essential to determine if the observed radiographic precision translates into improved clinical outcomes and implant longevity.

## Conclusion

The KUNWU open-platform robotic-assisted THA system demonstrated excellent radiological accuracy in this initial cohort. Mean deviation from the planned acetabular position to post-operative CT measurements was minimal (1.0° for version and −2.7° for inclination). Furthermore, 80.5% of all acetabular cups were placed within the combined safe zones of Callanan and Lewinnek. These results support the use of this novel system as a precise tool for accurately positioning acetabular components. Future studies with larger cohorts and direct comparisons to other platforms are warranted to further validate its efficacy.

## Data Availability

No datasets were generated or analysed during the current study.

## Abbreviations

THA: Total hip replacement
LLD: Leg length discrepancy
CT: Computed tomography
AI: Acetabular inclination
AV: Acetabular version
AO: Acetabular offset
COR: Centre of rotation
ICC: Intra-class correlation
CI: Confidence interval
AP: Anterior-posterior
